# Human and environmental controls on soil contamination in a dust-prone region revealed by random forest and Shapley additive explanations analysis

**DOI:** 10.1038/s41598-026-40377-x

**Published:** 2026-02-21

**Authors:** Zohre Ebrahimi-Khusfi, Shamsollah Ayoubi, Seyed Arman Samadi-Todar, Narjes Okati

**Affiliations:** 1 Department of Environmental Sciences and Engineering, Faculty of Natural Resources, University of Jiroft, Jiroft, Iran; 2 Department of Soil Science, College of Agriculture, Isfahan University of Technology, Isfahan, 8415683111 Iran; 3 Department of Remote Sensing, Faculty of Geographical Sciences, University of Tehran, Tehran, Iran; 4 Department of Environment, Faculty of Natural Resources, University of Zabol, Zabol, Iran

**Keywords:** Soil pollution, Human activities, Machine learning, Land degradation, Remote sensing, Arid regions, Environmental sciences, Natural hazards

## Abstract

Accurate prediction of the spatial distribution of potentially toxic elements (PTEs) and identification of the most important environmental drivers are essential for reducing their adverse effects on human health and the environment. In this regard, the present study was conducted to predict the spatial distribution of arsenic (As), cadmium (Cd), cobalt (Co), chromium (Cr), and lead (Pb) in a dust-prone area of central Iran using the RF model under 11 scenarios constructed based on human activity-based factors (HAF), land-based factors (LSF), physicochemical soil properties) PSP), meteorological factors (MF), and remote sensing auxiliary data (RSAD). The overall contribution of the influencing factors in predicting soil PTEs was determined using the SHapley additive exPlanations (SHAP) analysis. Soil PTEs and some other properties of 107 surface soil samples were measured in the laboratory. The best performance of RF in predicting As, Co, and Cr was observed under scenario VI (HAF + PSP) with the R^2^ value of 0.59, 0.60, and 0.58, respectively. The RF under Scenario X (PSP + LSF+HAF+RSAD) showed the best performance in predicting Cd (R^2^ = 0.67). The performance of RF for predicting Pb was weak in all scenarios (R^2^ < 0.38). The contributions of HAF, LSF, PSP, and RSAD in predicting Cd were 54.9, 21.5, 18.3, and 5.2%, respectively. On average, the contributions of HAF and PSP to the prediction of the other three PTEs were 55.6 and 44.4%, respectively. Among these categories, distance to industries, calcium, magnesium, magnetic susceptibility, terrain ruggedness index, and distance to rivers were identified as the most important predictors. Our findings are useful for improving soil management to reduce the adverse effects of PTEs in arid environments.

**Supplementary Information:**

The online version contains supplementary material available at 10.1038/s41598-026-40377-x.

## Introduction

Potentially toxic elements (PTEs), which are considered dangerous environmental pollutants, are toxic, persistent, and non-biodegradable^1,2^. Various factors like geological formations, industrial activities, and mining and agricultural practices affect their spatial variability^3,4^.

Contamination from the accumulation of these elements in soil can lead to land degradation^5^and affect food security and sustainable development in various regions, especially in arid and semi-arid areas^6–8^. The entry of PTEs into the human body through various means such as water, air, food, and even occupational exposure is toxic to the functioning of body cells and poses a serious threat to human health^9–11^.Therefore, identification of PTE-contaminated areas is a critical pre-condition for more efficient and strengthened administration of contaminated areas and reducing the risks of pollution, especially in dusty areas. Soils of such places are more susceptible to wind erosion, and soil erosion is harmful not only to nearby ecosystems and residents but also to distant places^12–14^. Numerous studies in Iran have shown that PTEs such as arsenic (As), cadmium (Cd), chromium (Cr), nickel (Ni), lead (Pb), and other metals in desert and dust-prone soils can pose serious risks to human health^15,16^. Therefore, uncovering the spatial distribution patterns of soil PTEs and identifying contaminated areas, especially in sensitive and vulnerable regions, is essential. These actions will contribute to better land management, ensuring food security, preserving ecosystem balance, and improving human quality of life.

Accurate PTEs prediction and determination of the controlling factors’ role are crucial for contaminated-soils hazard mitigations in different regions^17–19^. In recent decades, machine learning algorithms (MLAs) have gained attention as powerful tools for predicting and modeling complex spatial and temporal patterns of soil PTEs^20–23^.

Various MLAs have shown region-specific effectiveness in predicting potentially toxic elements (PTEs). RF worked well for Cu, Pb, and Zn globally^24^, and for Ni and Cu in western Iran, while Cubist excelled in Mn prediction^25^. RF also showed strong performance for Zn, Mn, Ni, Cr, Co, and Cu in central Iran^26^, and for As at a brownfield site^27^. SVR with RBF kernel outperformed ANN for predicting multiple PTEs in central and northern Iran^28^. Hybrid ANN models were superior to standalone ANN for Cu, Mn, and Ni in Russia^29^. In eastern China, RF and SVM surpassed ANN for As, Zn, Pb, Hg, Cu, and Cd^30^, while in South Korea, CNN with autoencoder outperformed RF and ANN for As, Cu, and Pb^31^.

Selecting the best combination of predictor variables is one of the most effective factors on the accuracy of predicting PTEs, which has been addressed in some studies. For instance, in a study conducted to predict the content of Zn, Mn, Fe, Co, Cr, Ni and Cu in Iran based on the RF model in three scenarios, the highest performance was reported in the third scenario, which included topographic factors, remote sensing data and soil properties^26^. However, in different studies various combination of environmental auxiliary variables have been introduced in modeling^32^.

The arid regions of central Iran, and especially Yazd province, are highly susceptible to wind erosion and dust storms, which contribute to the further dispersion of PTEs. Despite the significant consequences for human health, especially through inhalation and occupational exposure routes, these vulnerable areas have received limited targeted scientific attention. In this study, the spatial variability of HMs was assessed for the first time in this region through simultaneous evaluation using RF and various scenarios incorporating different environmental and anthropogenic factors were considered to identify key contributors to HM distribution patterns. Therefore, the present study was conducted to fill the mentioned research gaps through the following primary objectives: (i) to assess the predictive performance of the RF model for five soil PTEs under 11 scenarios constructed based on a set of human activity-based factors (HAF), land-based surface factors (LSF), physicochemical soil properties (PSP), meteorological factors (MF), and remote sensing auxiliary data (RSAD); (ii) to quantify the overall contribution of each factor to the spatial variability of soil PTEs using the SHAP analysis; and (iii) to identify the key environmental drivers and elucidate their mechanisms of influence on the spatial distribution of soil PTEs within a dust-prone region of central Iran.

## Materials and methods

### Study area

**Fig. 1:**
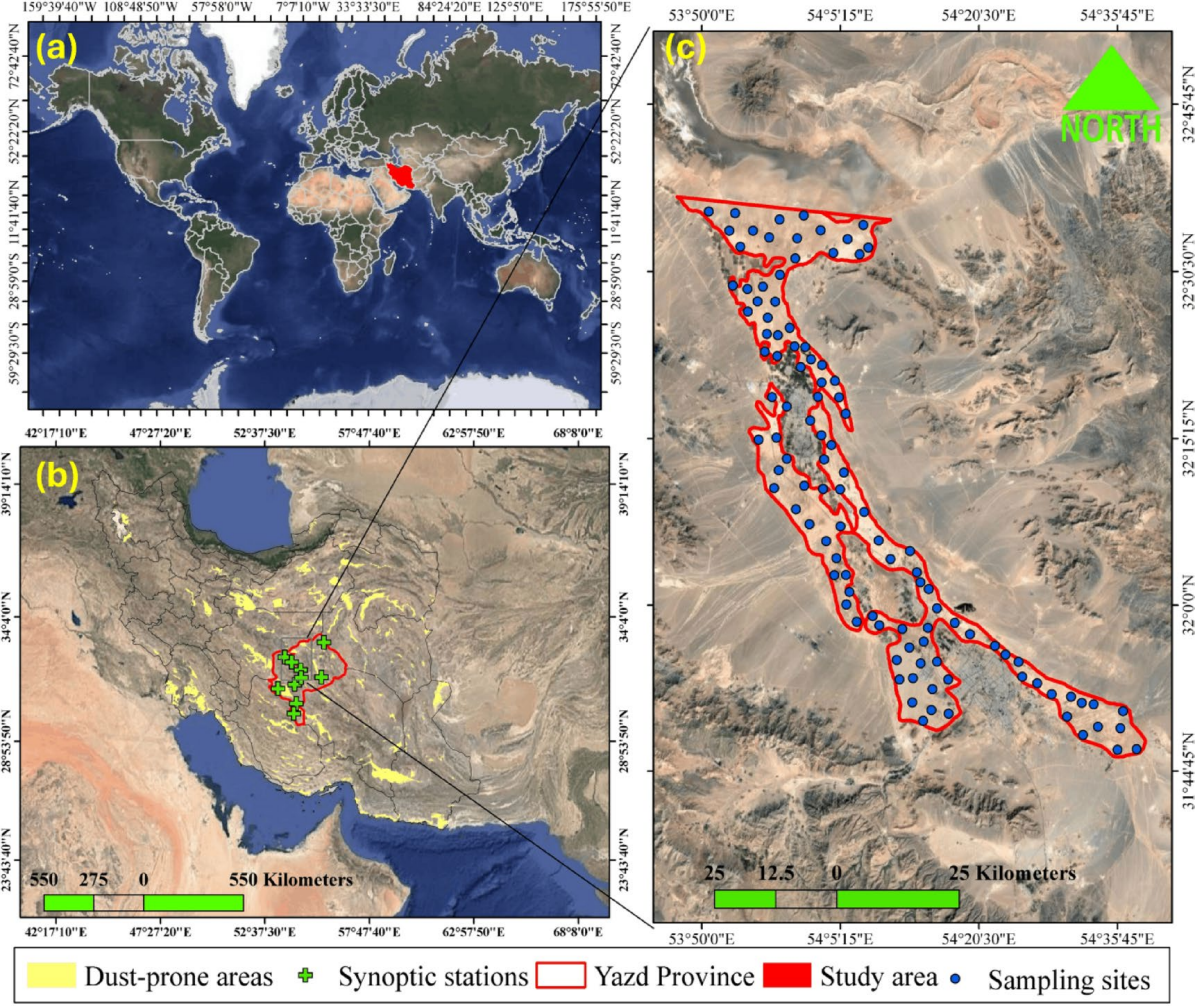
(**a**) Geographical location of Iran within the global context; (**b**) distribution of synoptic stations and dust-prone areas in Yazd province.; (**c**) location of sampling sites within the study area. Maps created using the free and open-source software QGIS 3.40.12-Bratislava (QGIS Development Team; https://qgis.org/download/).

The research’s study area is a dust-prone zone in the central parts of Yazd Province and the Central Iranian Plateau, stretches across longitudes 53° 49’ 10” E to 54° 34’ 40” E and latitudes 31° 50’ 20” and 32° 35’ 50” N (Fig. 1). This area spans 1057.1 km^2^ and average elevation of about 1170 m above sea level. Based on the Köppen-Geiger climate classification system^33^, it has a predominantly hot desert climate. Based on long-term meteorological statistics recorded at synoptic stations located in this area, the long-term average temperature, precipitation, and evapotranspiration are 21.5°C, 54.7 mm, and 3258 mm, respectively. The predominant direction of dusty winds is mainly from the north and northwest^34^. This region boasts high geological diversity, featuring formations dating from the Precambrian to the Holocene^20^. The dominant soil great groups over the study area included Typic Haplosalids, Typic Torriorthents, Typic Haplocalcids, Typic Haplogypsids, and Typic Torripsamments, which belong to the soil orders Entisols and Aridisols^35^. According to the World Reference Base (WRB), these soils correspond to Calcisols, Gypsisols, and Regosols.

### Soil sampling and analysis of PTEs and some soil properties

Soil sampling was conducted in January and February 2025, and 107 surface soil samples (0–5 cm) were collected from the study area (Fig. 1). After the soil samples were transferred to the laboratory, any remaining straw, stubble, and gravel were removed, and the samples were air-dried before being passed through a 2 mm sieve. Two grams of each sample were digested by aqua regia solution an finally the concentrations of five selected PTEs included As, Cd, Co, Cr, and Pb were measured using an Inductively Coupled Plasma-Optical Emission Spectrometry (ICP-OES) instrument^36^. For quality assurance and quality control, all equipment and containers were soaked in 10% HNO3 for 24 h and then rinsed thoroughly in deionized water before use. Each soil sample was replicated three times during digestion and analyses. Qtest was used for the identification and rejection of outliers and was applied to exclude abnormal readings at a confidence level of 95%. Moreover, a standard reference sample (San Joaquin #2709, National Institute of Standard and Technology, USA)) was employed for quality assurance in HMs analyses.

The volumetric method and the Walkley-Black method were used to measure calcium carbonate equivalent (CCE) and organic matter (OM) in soil samples, respectively^37,38^. Electrical conductivity (EC) was determined using the saturation extract method, and sodium adsorption ratio (SAR) was calculated based on soluble cation concentrations (Na⁺, Ca²⁺, Mg²⁺) in the extract^39–41^. Soil texture was then determined using the hydrometer method^42^. The magnetic susceptibility at both high frequency (χhf; 0.46 kHz) and low frequency (χlf; 4.6 kHz) was determined using a dual-frequency sensor (Bartington MS2). The frequency-dependent susceptibility (χfd%) was calculated using Eq. (1)^26,43^:

1 $$\:\chi\:f\mathcal{d}\left(\mathcal{\%}\right)=\left[\frac{\chi\:lf-\chi\:hf}{\chi\:lf}\right]\times\:100$$

### Target and predictor variables

In the present study, the concentrations of five PTEs were considered as target variables. Five groups of predictor variables were used to predict the concentrations of soil PTEs within the study area. These groups included (1) physicochemical soil properties (PSP; *N* = 13), (2) land-based surface factors (LSF; *N* = 21), (3) human activity-based factors (HAF; *N* = 10), (4) meteorological factors (MF; *N* = 4), and (5) remote sensing auxiliary data (RSAD; *N* = 21). All raster layers related to the predictor variables were resampled to 30 m by 30 m, matching the largest pixel size, which belonged to topographic properties.

#### Physicochemical soil properties (PSP)

The soil physicochemical properties used to predict PTEs included CCE, OM, EC, Na⁺, Ca²⁺, Mg²⁺, SAR, sand, silt, clay, pH, and χfd (%). The soil moisture was generated using the OPRTRAM-2 model derived from landsat-8 imageries^44^. The spatial distributions of PSP were shown in Fig. S1.

#### Land surface factors (LSF)

Topographical factors, lithological formations (LF), land use (LU), surface vegetation cover, bare soil index (BSI), modified normalized difference water index (MNDWI), and land surface temperature (LST) are among the other effective factors in predicting PTEs concentration^20,21,32,45,46^which have been considered as LSF in the present study. The 13 topographic factors used to predict concentrations of PTEs were digital elevation model (DEM), Aspect, slope, Topographic Position index (TPI), Topographic Ruggedness Index (TRI), Topographic Wetness Index (TWI), flow accumulation (FA), analytical hill-shading (AH), longitudinal curvature (LH), plan curvature (PLC), profile curvature (PRC), wind exposition (WE), and wind shelter index (WSI). The NASA SRTM Digital Elevation 30 m image (USGS/SRTMGL1_003) was obtained for the study area using the Google Earth Engine platform (https://earthengine.g.oogle.com) and other topographic factors were prepared using the SAGA GIS^47^.

In this study, the LU map was obtained from the General Department of Natural Resources and Watershed Management of Yazd Province, and the LF map was acquired from the Geological Survey and Mineral Explorations of Iran. LST, BSI, MNDWI, Soil Adjusted Vegetation Index (SAVI), MSAVI2, Normalized Difference vegetation Index( NDVI), and enhanced vegetation index (EVI) maps were generated using the Landsat-8 imageries for the study area in the Google Earth Engine platform(https://earthengine.google.com). The spatial distribution of these factors was shown in Fig. S2.

#### Human activity-based factors (HAF)

Rapid population growth, increased waste production, the expansion of industrial areas, mining activities, and transportation systems construction are among the key factors contributing to the increased concentration of PTEs^48–51^. Since industries can have a broader impact on soil contamination due to the emission of gases and suspended particles, while mines mainly act as local and point sources of pollution, this study treated the distance from industries and mines as two separate parameters^52,53^. This approach was adopted to better reveal the effect of each source on the spatial distribution of soil contamination in the region. Distance from rivers is also considered one of the predictive variables for the concentration of PTEs in soil^20,45^. Therefore, spatial layers representing the distance to residential areas, waste disposal sites, industrial sites, mines, and roads were generated using the Euclidean distance method in ArcGIS 10.8, with the same spatial resolution as the other factors (30 m×30 m). The spatial distributions of these factors were shown in Fig. S3.

#### Meteorological factors (MF)

Given that meteorological factors also control the distribution of the PTEs in soil^32,54^, these factors were also considered in modeling. The monthly values of four meteorological elements, temperature, precipitation, evaporation, and wind speed, for synoptic stations located in Yazd Province were obtained from the Iranian Meteorological Organization. The long-term average values of the mentioned climatic factors for the sampling season (winter) were calculated and spatial variation maps of these factors were prepared (Fig. S4).

#### Remote sensing auxiliary data (RSAD)

In many previous studies, the important role of the spectral bands of satellite imagery has been proven for predicting the concentration of PTEs^25,26,55^. The RSAD used to predict PTE concentrations in this study included the reflectance values of Landsat 8 sensor bands 2 to 7, which were downloaded for the time period corresponding to the sampling date. Also, using these bands, several band ratios were produced in the Google Earth Engine platform(https://earthengine.g.oogle.com), and were used to predict soil PTEs. The spatial distributions of RSAD were shown in Fig. S5.

### Modeling

#### Multicollinearity analysis and Scanrio defintion

In the regression analysis, the variance inflation factor (VIF) and tolerance coefficient (TC) are used to investigate the intensity of collinearities between independent factors^56^. The VIF values greater than 10 suggests that independent variables are strongly correlated and should be excluded to reduce modeling and prediction errors^26,57^. In this study, the VIF (Eq. 2) was used to identify multicollinearity among the predictor variables.

2 $$\:VIF=\frac{1}{1-{Ri}^{2}}$$

Here, Ri^2^ denotes the unadjusted coefficient of determination for the ith independent variable regressed against the remaining variables.

After removing highly correlated variables (VIF > 5), several scenarios were constructed. In the present study, five main groups of environmental variables are considered: PSP, LSF, HAF, RSAD, and MF. Scenarios were constructed by choosing one group as the base and then adding progressively the other groups. Using PSP as the base group, for instance, some scenarios include only PSP, PSP + LSF, PSP + LSF+HAF, PSP + LSF+HAF+RSAD, and PSP + LSF+HAF+RSAD + MF (Table 1). The same procedure was applied to the other groups, with the exception of MF, for which scenario construction was not carried out because there was a limited number of variables in it. After removing duplicate scenarios from the initial 20, 11 unique scenarios were modeled. This ensures all logical combinations are covered without unnecessary repetition.

**Table 1:** Definition of study scenarios based on the integration of physicochemical soil properties (PSP), land surface factors (LSF), human activity-based factors (HAF), remote sensing auxiliary data (RSAD), and meteorological factors (MF). The bolded scenarios represent the selected scenarios.

Scenarios	
PSP	LSF
**PSP + LSF**	LSF + PSD
**PSP + LSF+HAF**	LSF + PSD+HAF
**PSP + LSF+HAF+RSAD**	LSF + PSD+HAF+RSAD
**PSP + LSF+HAF+ RSAD + MF**	LSF + PSD+HAF+ RSAD + MF
**HAF**	**RSAD**
**HAF + PSP**	**RSAD + PSP**
HAF + PSP+LSF	**RSAD + PSP+LSF**
HAF + PSP+LSF+RSAD	RSAD + PSP+LSF + HAF
HAF + PSP+LSF+RSAD + MF	RSAD + PSP+LSF + HAF+MF

#### RF modeling

The RF algorithm is one of the most powerful and widely used MLMs, which is used for both classification and regression problems^58,59^. In this algorithm, first a subset of training data is randomly selected and for each decision tree, a subset of features is randomly selected. A decision tree is then built on this subset of data and features. For a new instance, each decision tree makes one prediction. The final prediction of the random forest is determined based on the majority of trees (in classification) or the average of tree predictions (in regression). The accuracy of this method largely depends on the number of trees generated^60^. This algorithm stands out due to its high accuracy, reduced overfitting, robust handling of large datasets, its capacity to reveal feature importance, and strong resilience to outliers^61–63^, which has caused it to be considered in many soil science studies^25,26,64,65^. Therefore, in this study, this algorithm was used to predict PTEs based on 11 selected scenarios.

The RF model’s output (y) is represented as follows:

3 $$\:y=\frac{1}{{n}_{tree}}\sum\:_{i=1}^{ntree}{f}_{i}\left(x\right)$$

Here, f_i_(x) refers to the discrete predictive output of a tress for x.

To effectively deal with spatial autocorrelation and overly optimistic model performance, model tuning and validation were done using spatially blocked cross-validation. The samples were spatially split into independent blocks, with about 80% being used for tuning and 20% remaining for spatially independent validation^66^. By doing so, it ensures that samples used for tuning and validation are spatially separate.

#### Performance assessment and optimal scenario selection

For the performance analysis of modeling, the three criteria of R^2^, root mean square error (RMSE), and mean absolute error (MAE), were employed as follows:

4 $$\:{R}^{2}={\left[\frac{n{\sum\:}_{i}^{n}{Me}_{i*}{Pr}_{i}-{\sum\:}_{i}^{n}{Me}_{i}{\sum\:}_{i}^{n}{Pr}_{i}}{\sqrt{n{\sum\:}_{i}^{n}{Me}_{i}^{2}-({\sum\:}^{n}{Me}_{i}{)}^{2}}\sqrt{n{\sum\:}_{i}^{n}{Pr}_{i}^{2}-({\sum\:}^{n}{Pr}_{i}{)}^{2}}}\right]}^{2}$$

5 $$\:RMSE=\sqrt{\frac{1}{n}{\sum\:}_{i}^{n}({Me}_{i}-{Pr}_{i}{)}^{2}}$$

6 $$\:MAE=\frac{{\sum\:}_{i=1}^{n}\left|{Me}_{i}-{Pr}_{i}\right|}{n}$$

where n is the total number of variables in a given dataset. The Me_i_ and Pr_i_ are measured and predicted values ​​of PTEs, respectively.

At the final stage of the present study, the model-scenario with the highest R^2^ and the lowest error statistics was selected as the optimal model-scenario for generating the predicted map of the PTE under investigation. Subsequently, to identify high-risk areas (hotspots) in the generated maps, the Getis-Ord Gi* statistic was applied^67^. In this method, pixels with values above the 95th percentile and a p-value less than 0.05 were considered hotspots. These analyses were conducted in R4.4.1 using the terra, sf, spdep, and tidyverse packages.

#### Importance of predictive variables and model explainability

Determining the contribution and impact of key factors on changes in the concentration of PTEs is essential for better management and reduction of the negative effects of soil pollution. In the present study Shapley Additive exPlanations (SHAP) method was used to explain how each feature (or input) influences the predictions of RF model^68^. Generally, variables with larger absolute SHAP values are recognized as more influential factors in predicting the PTEs. SHAP value is computed as follows:

7 $$\:{\phi\:}_{i}=\sum\:_{S\subseteq\:N\setminus\:\left\{i\right\}}\frac{\left|S\right|!\left(\left|N\right|-\left|S\right|-1\right)!}{\left|N\right|!}\left[f\left(S\bigcup\:\left\{i\right\}\right)-f\left(S\right)\right]$$

$\:\mathrm{H}\mathrm{e}\mathrm{r}\mathrm{e},\:{\phi\:}_{i}$ is importance of variable i in predicting PTEs. N is the entire set of predictor variables and f(S) denote the model’s PTE prediction given only the variables in set S. Also, $\:f\left(S\bigcup\:\left\{i\right\}\right)$ refers to model output when variable i is added to set S.

In the present study, in addition to determining the individual contribution of each environmental variable, SHAP interaction values were calculated for the key features of each group to assess their combined role in predicting the concentrations of PTEs.

## Results and discussion

### Descriptive statistics

**Fig. 2:**
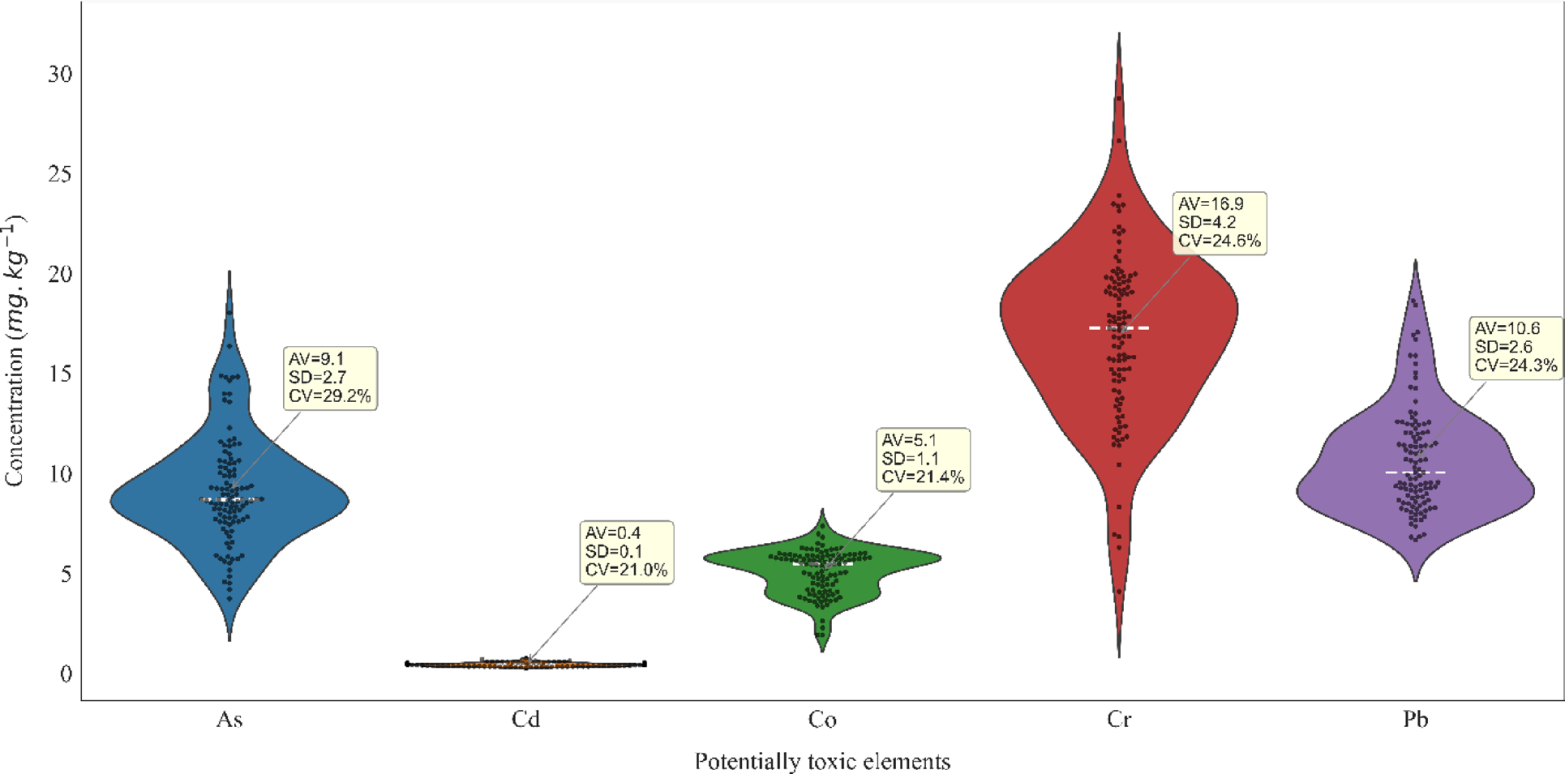
Statistical characteristics of potentially toxic metal concentrations in the study area, including mean (AV), standard deviation (SD), and coefficient of variation (CV).

The statistical characteristics of the PTEs studied in the research area are shown in the box plots in Fig. 2. The highest average concentrations in the study area belong to Cr (16.9 mg/kg), followed by Pb (10.6 mg/kg), As (9.1 mg/kg), Co (5.1 mg/kg), and Cd (0.4 mg/kg). The average concentrations of As, Cd, Co, Cr, and Pb in the Earth’s crust are reported as 1.8, 0.15, 25, 102, and 14 mg/kg, respectively^69^. The contents of As and Cd are 7.3 and 0.28 mg/kg higher than the Earth’s crust, while the other PTEs are below the crustal values. The crustal enrichment of As and Cd might be due to natural factors such as specific mineralization in the parent rocks or anthropogenic activities. Mean concentrations of Pb, As, and Cd in the southern parts of the study area were reported higher than background concentrations^70^, which supports the findings of this study.

The concentrations of As and Cd are lower than in Lake Urmia deposits in northwestern Iran^71^and Hamoun Wetland in southeastern Iran^15^. The concentrations of Cr and Co are lower than the sites mentioned in (Table 2) around the Iran. Although the concentration of Pb in the study area’s soils is lower than in the soils examined in the three provinces of Tehran^72^, Yazd^73^, and West Azerbaijan^71^, it is higher than in selected soils of Khuzestan^74^and the Sistan Plain^75^. These differences can be attributed to variations in the number of samples, sampling depth and time, and the different impacts of human and environmental factors in various regions. In terms of the coefficient of variation (CV), based on the classification by Wilding^76^, all study PTEs fall into the moderate-variability class (15% to 35%), indicating that heir spatial distribution is likely influenced by natural processes^25,26^or widespread, diffuse anthropogenic activities.

**Table 2:** Mean concentrations (mg/kg) of potentially toxic elements (PTEs) in the study area other parts of Iran.

Study regions	As	Cd	Co	Cr	Pb	Reference
Tehran Province	-	0.13			21.3	^72^
Isfahan Province	-	-	20.2	54.3	-	^26^
Sistan Province	37.7	0.4	8.3	39.1	10.1	^75^
Yazd Province	-	0.26	-	-	40.9	^73^
Weiss region-Khuzestan Province	-	1	8	20.1	9	^74^
Arab Asad- Khuzestan Province		1	9.1	21.1	9	
Lake Urmia	10	0.24	-	83	22	^71^
Yazd Province	9.1	0.4	5.1	16.9	10.6	This study

### Performance assessment and optimal scenario selection

Before the PTEs modeling and prediction process, VIF method^77^was used to select variables with the least multicollinearity effect (VIF < 5). In the present study, 69 environmental variables belonged to five groups (PSP, LSF, HAF, RSAD, and MF). The VIF analysis was conducted stepwise; at each step within a group, only the variable with the highest VIF was removed, and when the VIFs of all variables in that group dropped below 5, the VIF analysis for that group was stopped (Fig. S6).

Our analyses revealed that among the 69 variables examined, 35 variables have a severe multicollinearity effect, so they were excluded. Consequently, the variables presented in (Table 3) were ultimately selected for modeling. Some of these variables, such as χfd, AH, pH, OM, TPI, FA, PLC, PRC, TWI, LU, Dis.Str, and Dis.Mines, have also been chosen in previous studies for modeling PTEs^24,78,79^.

To ensure that critical variables were not removed during the modeling process, Spearman’s correlation test was used to assess the correlation between PTEs and the 32 excluded environmental variables. The results showed that the correlation coefficients ranged from − 0.53 to 0.42 (Fig. S7), indicating no strong significant correlation between these two sets of variables. In other words, this confirms that critical variables were not removed, and the information from these variables is largely preserved by the remaining variables due to high multicollinearity.

**Table 3:** Optimal variables for predicting potentially toxic elements in the study area.

Variable group	Optimal variables	Abbreviation (unit)	VIF
Soil physicochemical properties	Frequency-dependent susceptibility	χfd ( %)	2.5
	Electrical conductivity	EC (ds/m)	3.1
	Organic matter	OM (%)	2.1
	Calcium carbonate equivalent	CCE (%)	2.0
	Magnesium	Mg (meq/lit)	3.7
	Calcium	Ca (meq/lit)	4.7
	Potential hydrogen	pH	2.3
	Sand	Sand (%)	3.1
	Clay	Clay (%)	2.4
Land-based surface factors	Aspect	Asp (degree)	2.6
	Slope	Slp (%)	4.3
	Topographic position index	TPI	2.3
	Terrain ruggedness index	TRI	2.6
	Topographic wetness index	TWI	2.8
	Flow accumulation	FA	2.3
	Analytical hillshading	AH	4.4
	Longitudinal curvature	Long (m^− 1^)	2.7
	Plan curvature	PLC (m^− 1^)	1.6
	Profile curvature	PRC (m^− 1^)	1.7
	Wind exposition	WE	3.4
	Wind shelter index	WSI	4.2
	Land surface temperature	LST (°K)	4.8
	Lithological formations	LF	2.4
Human activity-based factors	Distance to streams /rivers	Dis.Str (Km)	2
	Distance to railways	Dis.Rail (Km)	2.4
	Distance to villages	Dis.Vil (Km)	2.1
	Distance to industrial centers	Dis.Indust (Km)	4.3
	Distance to mines	Dis.Mines (Km)	2.4
	Land use/cover	LU	1.7
Meteorological factors	Precipitation	Pre (mm)	2.3
Remote sensing auxiliary data	Band5	B5	2.5
	Band ratio (2/4)	BR24	2.7
	Band ratio (4/7)	BR47	2.4
	Band ratio (6/7)	BR67	1.6

**Fig. 3:**
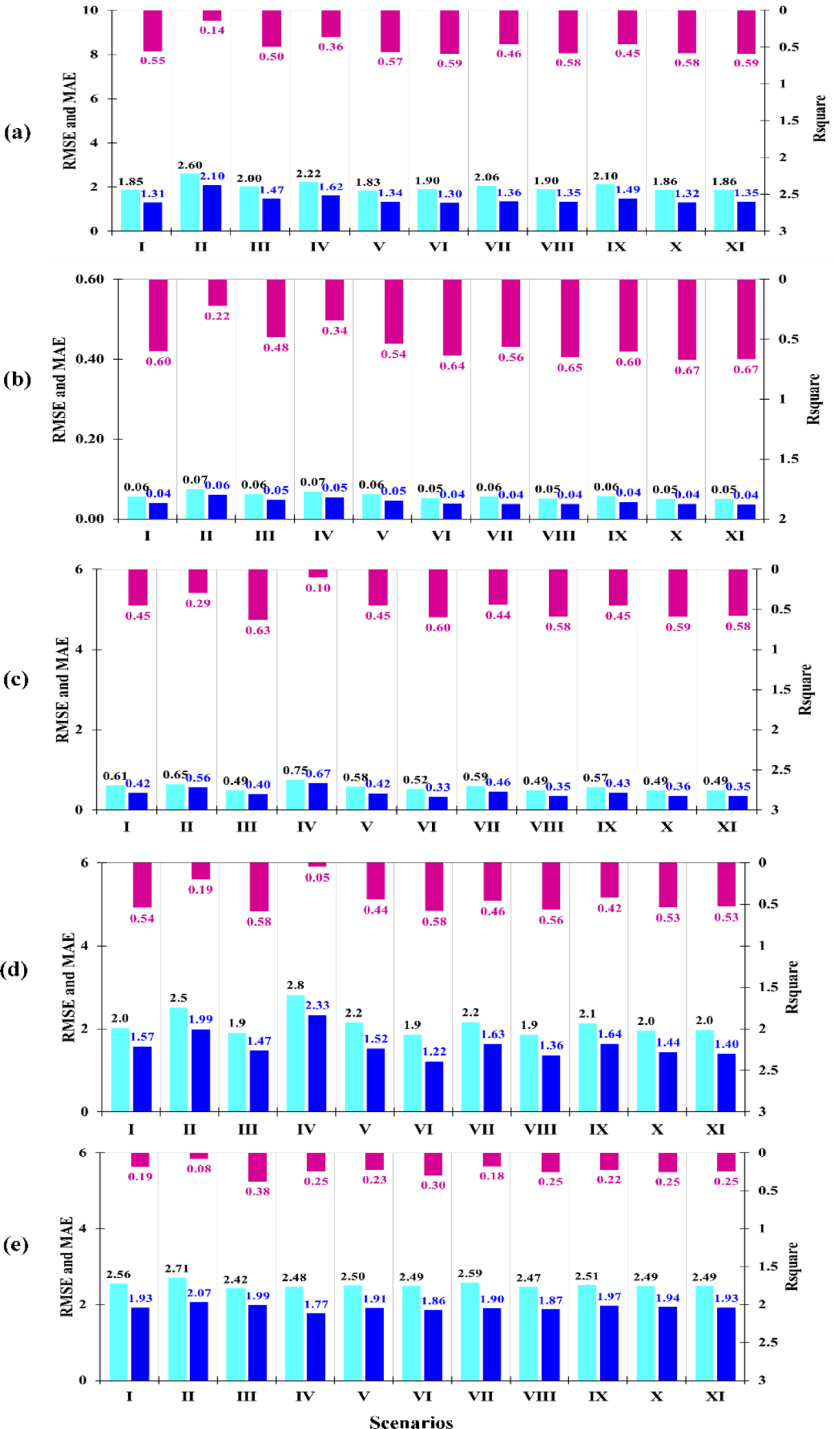
Performance assessment statistics for predicting (**a**) arsenic, (**b**) cadmium, (**c**) cobalt, (**d**) chromium, and (**e**) lead in the study area.

The results of the RF model’s performance evaluation for predicting each of the PTEs in the validation dataset are presented in Fig. 3. The evaluation was performed based on three statistical metrics: R^2^, RMSE, and MAE. The best performance of the RF model for predicting As (Fig. 3a) was observed using predictor variables from the scenario (VI), which included HAF + PSP (R^2^ = 0.59, RMSE = 1.90, MAE = 1.30). Meanwhile, the best performance of the model in predicting Cd was observed when the model was run with scenario (X: PSP + LSF+HAF+ RSAD) variables (Fig. 3b). In the validation dataset for this scenario, the values for R^2^, RMSE, and MAE were 0.67, 0.05, and 0.04, respectively.

The predictive performance for Co and Cr was maximized when the predictor variables from the scenario (VI: HAF + PSP), were used in the modeling process. Based on the validation dataset in the optimal scenario for predicting Co, the values for R^2^, RMSE, and MAE were obtained as 0.60, 0.52, and 0.33, respectively (Fig. 3c). In the selected scenario for Cr, the R^2^, RMSE, and MAE values were 0.58, 1.90, and 1.22, respectively.

The performance of the RF model for predicting Pb was evaluated as poor across all scenarios studied. As shown in Fig. 3d, the maximum R^2^ value, which was 0.38, belonged to scenario III. The minimum error statistics were estimated to be approximately 2.42 for RMSE and 1.99 for MAE. The failure of the RF model to accurately predict Pb concentrations in the arid study area, despite using comprehensive input variables, stems primarily from two issues: the high spatial skewness of the contaminant data (many low background values vs. few high hotspots), which RF struggles to fit, and the homogeneity of remote sensing data in dry environments, limiting the model’s ability to find strong splitting features. To address this, we propose two key improvements: log-transforming the Pb concentrations to normalize the skewed distribution, employing the XGBoost algorithm to iteratively focus on and correct prediction errors in the critical hotspot areas in future studies.

Hu, et al.^79^ also observed a very poor performance of RF (R^2^= 0.17) for predicting Pb in some selected soils of China, similar to what was observed in the present study for selected soils of the dust-prone zone in central Iran. The maximum R^2^ value estimated by the this model for predicting Fe, Zn, Mn, Cu, Cr, Co, and Ni in a part of Isfahan Province, Central Iran, was reported less than 0.42^26^. Although the land and environmental conditions of different regions vary, a part of this discrepancy might be related to human-based activities, which were not considered as predictor variables by these researchers in the three scenarios they examined. These results highlight the importance of predicting PTEs based on more scenarios.

Overall, the performance of RF model for predicting five PTEs based on the test dataset in the best scenarios was: Cd (R^2^= 0.67 *>* Co *>* As *>* Cr *>* Pb (R^2^= 0.38) (Fig. 3). Using this model and based on the R^2^ values, the highest predictive performance for the bioaccumulation factors of PTEs in China was observed for Zn (0.84), Cu (0.66), Cr (0.59), Ni (0.58), Hg (0.58), Cd (0.58), As (0.30), and Pb (0.17)^79^. Although among the four similar elements examined (Cd, Cr, As, Pb), the prediction accuracy for Cd in our study was higher than that of the other metals, in both studies, the prediction accuracy for Cr was higher than that for Pb.

Moreover, the current research confirmed high capability of the RF model in predicting the spatial pattern of distribution of the majority of soil PTEs, as confirmed in some previous researches in Iran^25,26^and globally^79–82^. The RF model’s effectiveness is attributed to its nature as an ensemble method, which allows it to accurately identify and model nonlinear relationships between the target and predictor variables^45,64^.

**Fig. 4:**
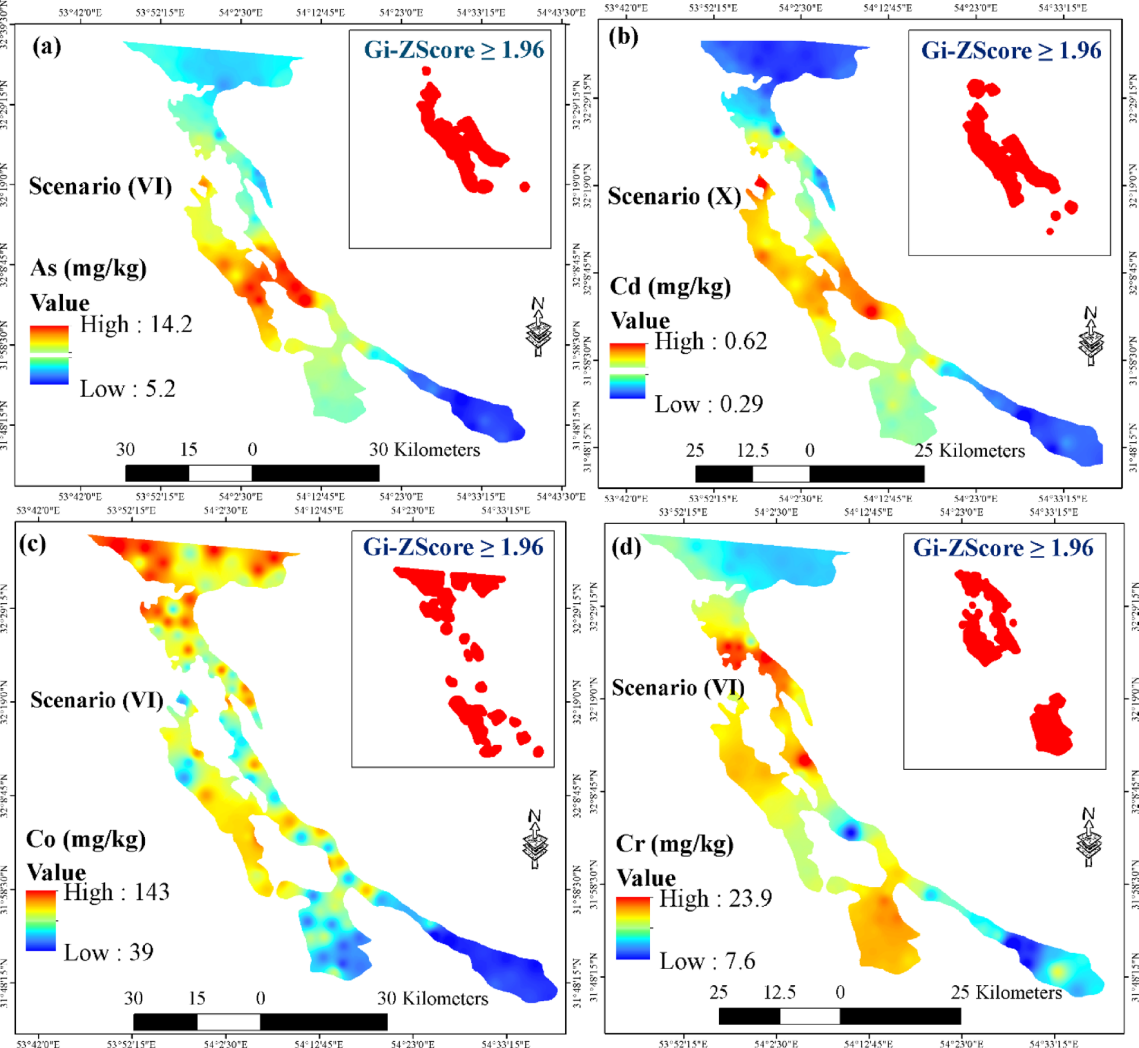
Spatial prediction maps of concentrations for (**a**) arsenic, (**b**) cadmium, (**c**) cobalt, and (**d**) chromium using the random forest model in the best-case scenario. Areas with Gi-ZScore ≥ 1.96 values represent hotspot locations that are statistically significant at the 95% confidence level. Maps created using the free and open-source software QGIS 3.40.12-Bratislava (QGIS Development Team; https://qgis.org/download/).

In the next stage, spatial prediction maps were generated using the RF model and predictor variables from the optimal scenario for four out of the five PTEs (As, Cd, Co, and Cr), whose good performance was confirmed in this study. The scatter plots of predicted versus measured values for these four elements in the best-case scenario are shown in Fig. S8, and their spatial distribution maps are displayed in Fig. 4 (a-d).

In the study area, the highest concentrations of As and Cd were found in the central and western regions. These critical areas are located near industrial zones and the Yazd-Tehran highway. In addition, Co and Cr primarily accumulate in parts of the northern areas, near an urban zone, and in the southwestern regions, close to a special economic zone. The similar spatial distribution pattern could indicate a common source for these elements in the aforementioned areas. Similar results were reported by Liang, et al.^83^ in southwestern China for As.

### Key factors and their influence on the spatial variability of soil PTEs

For diminishing the adverse effects of pollution caused due to soil PTEs accumulation, not only should the spatial distribution of these elements be precisely predicted but also the major environmental factors that control their concentration need to be identified^66,79^.

**Fig. 5:**
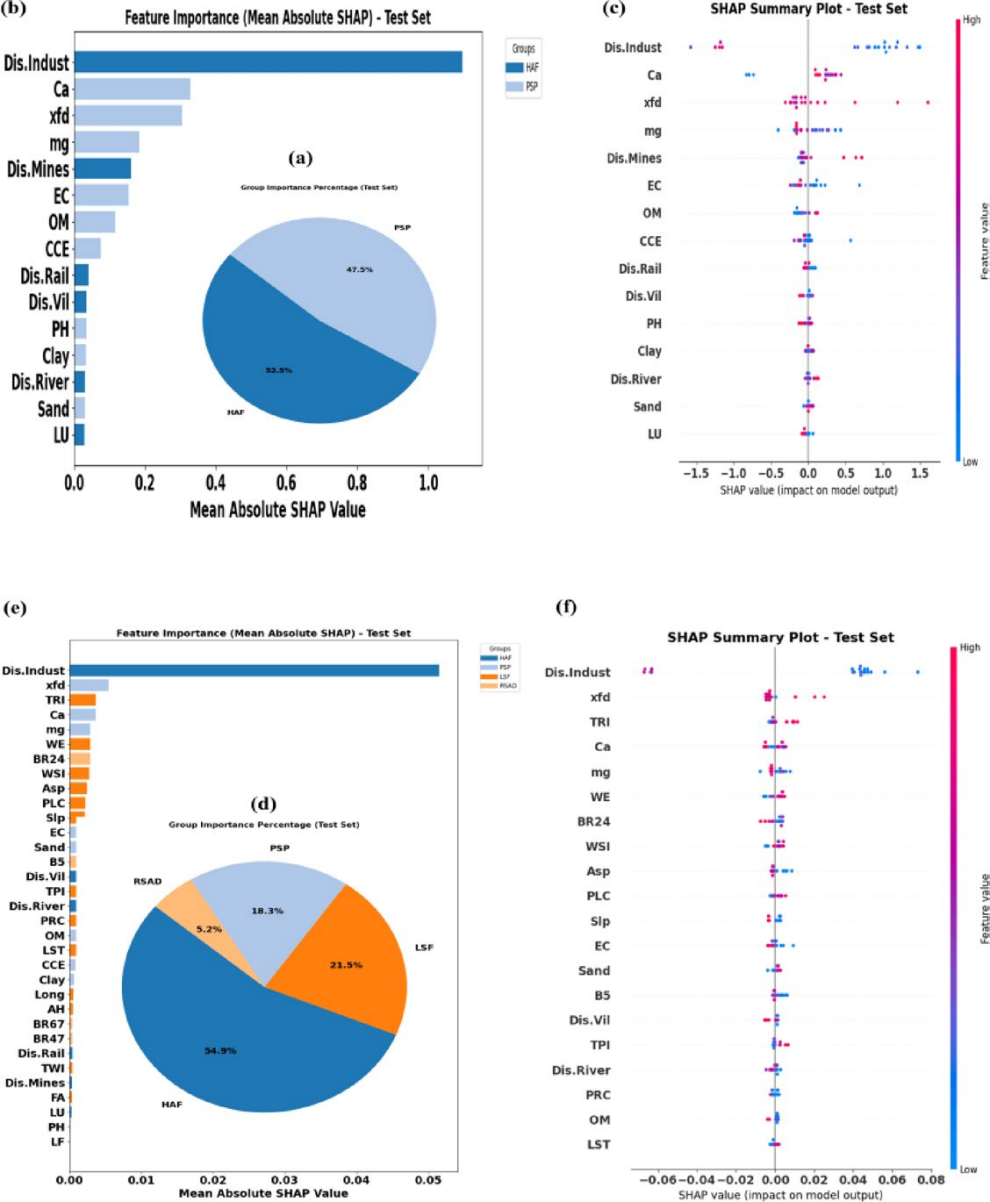

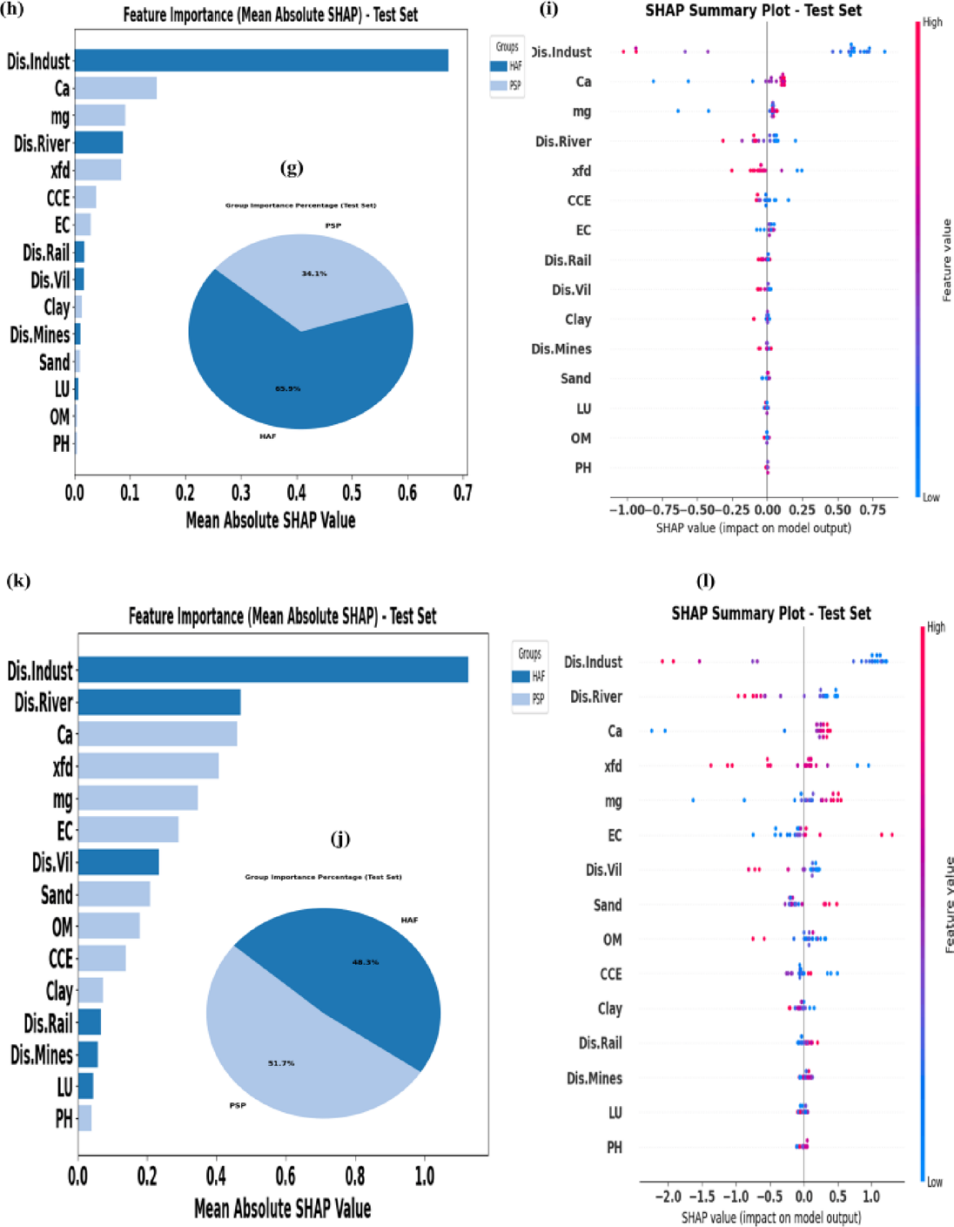
Key factors and their influence on the spatial variability of As (**a**–**c**), Cd (**d**–**f**), Co (**g**–**i**) and Cr (**j**–**l**) within the study area.

In the current study, key factors affecting the concentration of four elements whose acceptable performance was confirmed in the previous stage (As, Cd, Co, and Cr) were identified using the mean absolute SHAP values in the tuned model by optimal scenarios. The results are presented in Fig. 5 (a-l).

Based on the results, HAF had the greatest contribution in the distribution of three soil PTEs in the study area. The contribution of HAF to the spatial variability of Co, Cd, and As was 65.9% (Fig. 5d), 54.9% (Fig. 5a), and 52.5% (Fig. 5g), respectively. Although in some previous studies the average coefficient of variation of PTEs has been attributed to natural processes^25,26^, in this study it was observed that despite the average coefficient of variation of the studied PTEs, the contribution of human activities is high. This indicates that the pollution in this area, which is also prone to dust production, was mainly of the diffuse type, which increased the baseline level of PTEs, without creating severe heterogeneity in their spatial distribution.

Among the human-based activities, the relative importance of Dis.Indust was greater than other influencing factors. For the mentioned elements, mean absolute SHAP values were approximately 0.7 (Fig. 5e), 0.05 (Fig. 5b), and 1.1 (Fig. 5h), respectively. The share of this human-based factor is, however, varied for each element, a finding that has also been reported in some previous studies^32,81^. SHAP summary plot indicate that distance to industries is negatively correlated with the spatial pattern of some of the soil PTEs in the study area (Fig. 5c, f, i and l).

Soil PSP had also the greatest contribution in explaining the spatial changes of As (47.5%), Co (34.1%), and Cr (51.7%), in the study area. The Ca (Fig. 5b) and χfd (Fig. 5f) were identified as the second influencing factor for the spatial distribution of As (0.38) and Cd (0.008), respectively. The Ca ranked second in predicting Co (0.17, Fig. 5h) and Cr (0.4, Fig. 5k) concentrations. The SHAP summary plot further indicated that Ca had positive relation with distribution of soil As (Fig. 5c), Co (Fig. 5i), and Cr (Fig. 5l). For Cd, high SHAP values associated with χfd were scattered in both positive and negative directions (Fig. 5f), meaning that it has a multifaceted and complex influence on the distribution of this soil PTE. The distribution of high mg values in the negative range of the SHAP plot for As (Fig. 5c) and Cd (Fig. 5f) confirms an inverse relationship between them and mg. This is in contrast to Co (Fig. 5i) and Cr (Fig. 5l), where the maximum mg values are observed in the positive range, highlighting a direct relationship between these soil PTEs and mg in the study area. High contribution of mg as an element in soil with PTEs concentration, might be attributed to high tendency of these HMs to adsorb by Montmorillonite and Palygorskite minerals which are enriched by Mg in arid regions^84^. These findings indicate the relatively high importance of these two cations in predicting the concentration of soil PTEs in the study area. Our findings are consistent with those of Taghizadeh-Mehrjardi, et al.^20^, who reported the significant importance of cations in predicting PTEs concentrations in central Iran. Comparison of the spatial distribution of PTEs with soil properties (Fig.S1) showed the lowest concentrations were mainly observed in areas characterized by minimum clay content and maximum sand content. This pattern confirms the key role of clay particles in retaining the studied elements, particularly in their cationic forms. In contrast, special attention should be paid to areas with high silt content (Fig.S1), as these zones are highly susceptible to wind erosion. Given the arid conditions of the study area and its high potential for wind erosion, improper management may cause these areas to act as major source zones for particle entrainment. Consequently, suspended dust particles enriched with considerable amounts of PTEs may be released into the atmosphere, posing serious risks to human health.

The distribution of most high χfd values for As (Fig. 5c) and Cd (Fig. 5f) is observed on both sides of the SHAP plot. This indicates a complex and bidirectional influence of this factor on the distribution of As and Cd in the study area. In contrast, most of the points with high χfd values for Co (Fig. 5i) and Cr (Fig. 5l) are distributed within the negative range of SHAP values, which shows an inverse relationship between χfd and these two elements in the study area. Previous studies in northwest Iran reported a positive correlation between the two mentioned PTEs and the magnetic measures in low frequency^85^. For studied soils in the southwest of Iran, χfd showed a positive correlation with Co and a negative correlation with Cr^78^. Additionally, this important soil factor is mentioned as one of the important predictors of soil Co in central Iran^26^and confirms the findings of the present research. Therefore, it is a reliable predictor of cobalt variation, especially in Iran. The combination of PSP and magnetic susceptibility in explaining a high percentage of the variations in the concentrations of different soil PTEs has also been confirmed by Ayoubi, et al.^78^.

Although, based on the investigations conducted in the present study, land-based factors are not suitable for predicting Co and Cr, they are suitable for predicting the Cd. In some studies, conducted in Isfahan province, Iran^26^, and northern Guangdong province, China^86^, LSF has played an important role in explaining the spatial variations of some PTEs. However, in the studied soils, HAF played a more dominant role in explaining the distribution of soil PTEs. Therefore, for pollution control in our study region, a higher priority should be placed on managing and monitoring human-related pollution sources rather than on factors dependent on LSF. The contribution of LSF to the spatial variability of Cd was 21.5% (Fig. 5d). Among the LSF, the TRI factor, with a mean absolute SHAP value 0.003 had the greatest contribution in predicting the spatial distribution Cd (Fig. 5e). The SHAP analysis highlighted the positive impact of the TRI on the distribution of Cd in our study area (Fig. 5f). Also, the WE (0.005) had the low contribution in predicting Cd (Fig. 5e). The SHAP analysis indicated that this factor had a positive relation with spatial distribution of soil Cd, showing that higher values of WE were associated with higher accumulation of this element in the soil of the study area. The accumulation capacity of pollutants has been reported to be higher in some sheltered areas compared to exposed areas^87,88^, and a similar pattern was observed in our study area.

Although the contribution of some environmental controllers, such as LF to the prediction of Cd concentration is low, their role in this prediction should not be overlooked. For example; for Mur and Qs, the points are mostly located further from zero and in the negative range, indicating that these geological formations have reduced the prediction of Cd concentration. In contrast; the points for Qft2 and Qsf are located in the positive range, indicating their positive effect on the prediction of this potentially toxic element (Fig.S9).

The unique reflection and absorption of specific wavelengths by various metals in soils and rocks provide a valuable spectral fingerprint for detecting soil PTEs^89^. In the current study, it was also found that RSAD can be somewhat effective in explaining the spatial changes of Cd. The overall contribution of these factors to predict Cd in the soil of the study area was estimated at 5.2% (Fig. 5d). Different contributions of RSAD for explaining the spatial variability of some soil PTEs have also been reported by Azizi, et al.^25^ and Taghizadeh-Mehrjardi, et al.^20^. Among the remote sensing data, BR24 received the highest mean absolute SHAP value in predicting this element (Fig. 5e). The distribution of points with different values on the right side of the SHAP plot, along with a limited number of high-value points on the left side (Fig. 5f), demonstrates a complex relationship between the spectral reflectance of the BR24 and the spatial pattern distribution of Cd in the study region. Moradpour, et al.^26^ identified Landsat band 2 as a predictor for soil Cu, Cr, Co, and Ni. Accurate predictions of the spatial changes of Cd have been obtained by using the main bands of Landsat sensors in combination with other environmental predictors in northeastern Kazakhstan^90^and the Czech Republic^91^.Accordingly, and based on the findings of the present study, these types of predictors are suitable for analyzing the variability pattern of soil PTEs across different regions.

**Fig. 6:**
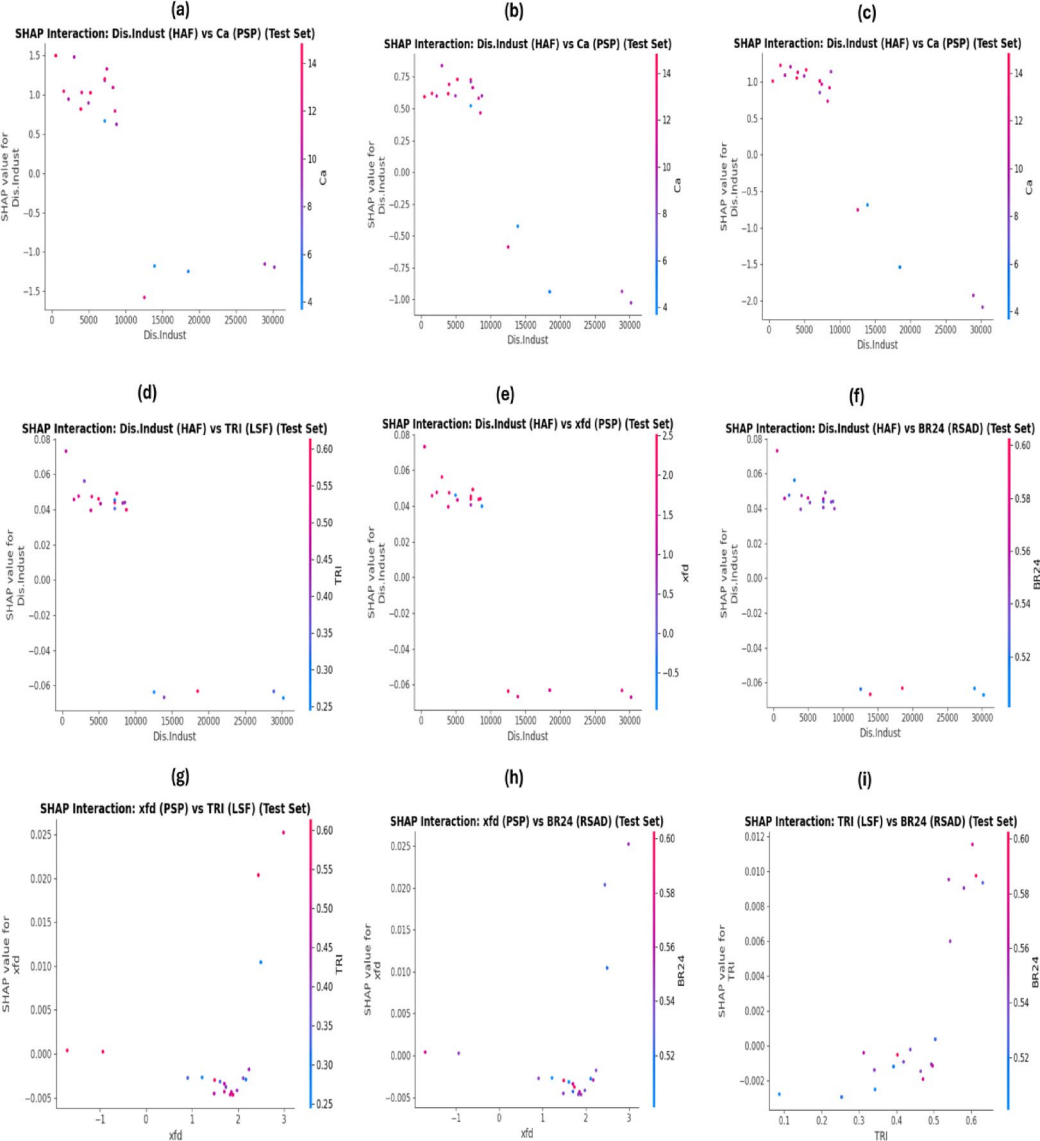
SHAP interaction analysis between the most important environmental variables for predicting (**a**) arsenic, (**b**) cobalt, (**c**) chromium, and (**d** to **i**) cadmium.

Based on the comparative interaction plots of the environmental variables with the highest contribution from HAF and PSP, it was found that near industrial areas, the increasing contribution of this human-driven factor to the prediction of As (Fig. 6a), Co (Fig. 6b), and Cr (Fig. 6c) rises in samples with higher Ca levels. This indicates a strong and reinforcing interaction between Dis.Indust and Ca in predicting the concentrations of these three PTEs in the study area.

For Cd prediction, the four variables Dis.Indust, χfd, TRI, and BR24 had the highest contributions from the HAF, PSP, LSF, and RSAD groups, respectively. As shown in the interaction plots, the interaction between Dis.Indust- χfd (Fig. 6d), Dis.Indust-TRI (Fig. 6e), and Dis.Indust-BR24 (Fig. 6f) is weak (SHAP = ± 0.06). A similarly interaction pattern was observed between χfd -TRI (Fig. 6g) and χfd -BR24 (Fig. 6h), as well as between TRI and BR24 (Fig. 6i), indicating that these environmental variables acted mainly independently and additively in predicting soil Cd concentration in the study area.

Overall, these findings show that the RF model, under optimized scenarios, was able to uncover the complex and hidden relationships among key environmental variables and how they influence the prediction of the concentrations of the four heavy elements in the study area.

## Environmental implication and limitations

The findings of this study have significant implications for management of contaminated and critical zones in a dryland part of central Iran. Central, western, and southwestern parts of the study region were identified as the most important hotspots which require special attention for pollution control and mitigation of adverse impacts.

In addition, identifying the overall contribution of key factors affecting the spatial distribution of As, Cd, Co, and Cr, along with their relative importance, is useful for better planning and management to reduce pollution resulting from the accumulation of these elements. This, in turn, can help mitigate land degradation in the study area. Specifically, industrial activities and certain soil physicochemical properties, such as Ca and ꭓfd, were identified as crucial indicators that can guide the prioritization of management and remediation measures in the study area.

Although the modeling and prediction process using multiple combined scenarios was time-consuming, it provided an appropriate framework for achieving more accurate predictions of spatial variability patterns of soil PTEs.

Nevertheless, several limitations should be acknowledged. One is the limited number of sampling points and the other is the use of an algorithm to predict the spatial distribution of PTEs in the study area. Also, due to the lack of access to certain data layers concurrent with the sampling period (such as land use and groundwater quality and quantity parameters), some potentially important variables were not included. Therefore, the future study is recommended to overcome these limitations, fulfill the research gaps available, and compare their result with the results of this current study.

## Conclusion

This study, for the first time, employed eleven scenarios to evaluate the performance of the RF model in predicting the spatial distribution of five PTEs in an arid environment in central Iran. The relative importance of environmental factors and their effects were also determined through SHAP analysis. The results showed that among 69 environmental variables, 34 variables with the lowest multicollinearity were suitable to predict PTEs in the study area. Model performance evaluation for the 11 scenarios indicated that Scenario VI (PSP + HAF) was best to predict As, Co, and Cr with R² values of 0.59, 0.60, and 0.58, respectively. In addition, the model was optimal under Scenario X (PSP + LSF+HAF+RSAD) for determining spatial variations of Cd with R² value of 0.67, whereas there were no acceptable outcomes for estimating soil Pb in the study area from any of the scenarios. Accordingly, other machine learning algorithms and alternative scenarios are recommended for more accurate prediction of soil Pb. The SHAP analysis revealed that HAF and PSP contributed most to the spatial distribution of four PTEs (As, Cd, Co, and Cr). Among HAF, industrial activities, and among PSP, variations in Ca and χfd were identified as key predictors. Two key factors, Dis.Indust and Ca, played a strong and synergistic role in predicting the concentration of As, Co, and Cr. In contrast, the interactions between the main predictive factors for Cd were additive and independent of one another.

Overall, the findings of this study confirmed that several scenario analyses can make more accurate predictions for the spatial distribution of most central Iran’s soil PTEs. Therefore, it is recommended in future research to study various scenarios for more precise predictions, particularly when soil sample numbers are limited. Moreover, inclusion of other easily proximal techniques such as portable X-ray fluorescence (pXRF) and other magnetic parameters like Remanent Magnetization, Anhysteretic Remanent Magnetization (ARM), and Isothermal Remanent Magnetization (IRM) should be evaluated as co-variables for predicting PTEs.

## Supplementary Information

Below is the link to the electronic supplementary material.

Supplementary Material 1

## Data Availability

The raw data are available from the corresponding authors upon reasonable request.
