# Supplementary material for: Human and environmental controls on soil contamination in a dust-prone region revealed by random forest and Shapley additive explanations analysis
Source: Sci Rep. 2026 Feb 21;16:10073. doi: 10.1038/s41598-026-40377-x (PMC13022290; doi:10.1038/s41598-026-40377-x)
Supplement: Supplementary file 1 — Supplementary Material 1 [file 41598_2026_40377_MOESM1_ESM.docx]

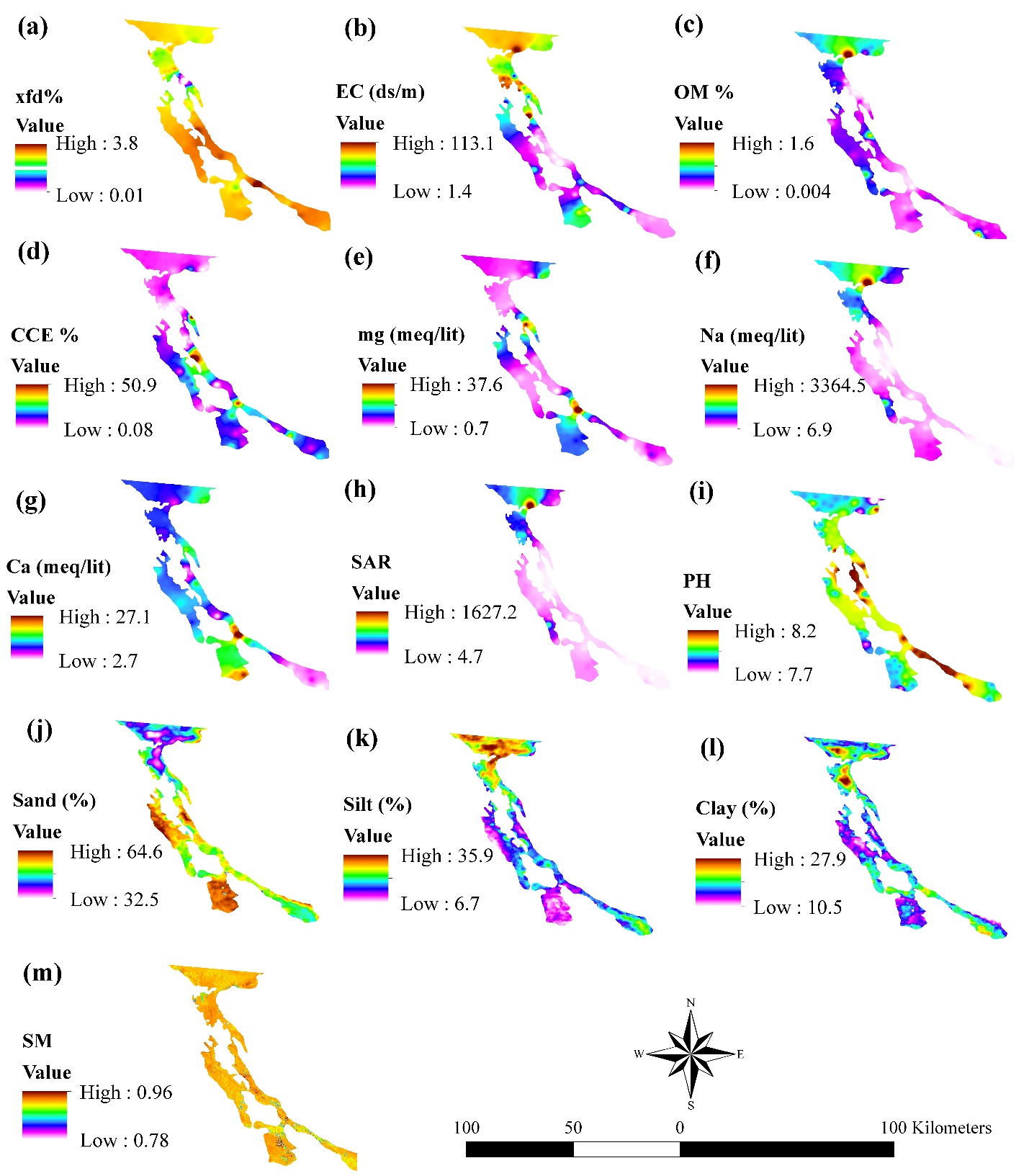


**Fig.S1.** Spatial distribution of soil physicochemical properties in the study area: (a) frequency-dependent susceptibility (χfd%), (b) electrical conductivity (EC), (c) organic matter (OM), (d) calcium carbonate equivalent (CCE), (e) Magnesium, (f) sodium, (g) calcium, (h) sodium absorption ratio, (i)  potential hydrogen (pH), (j) sand, (k)silt, (l) clay, and (m) soil moisture.


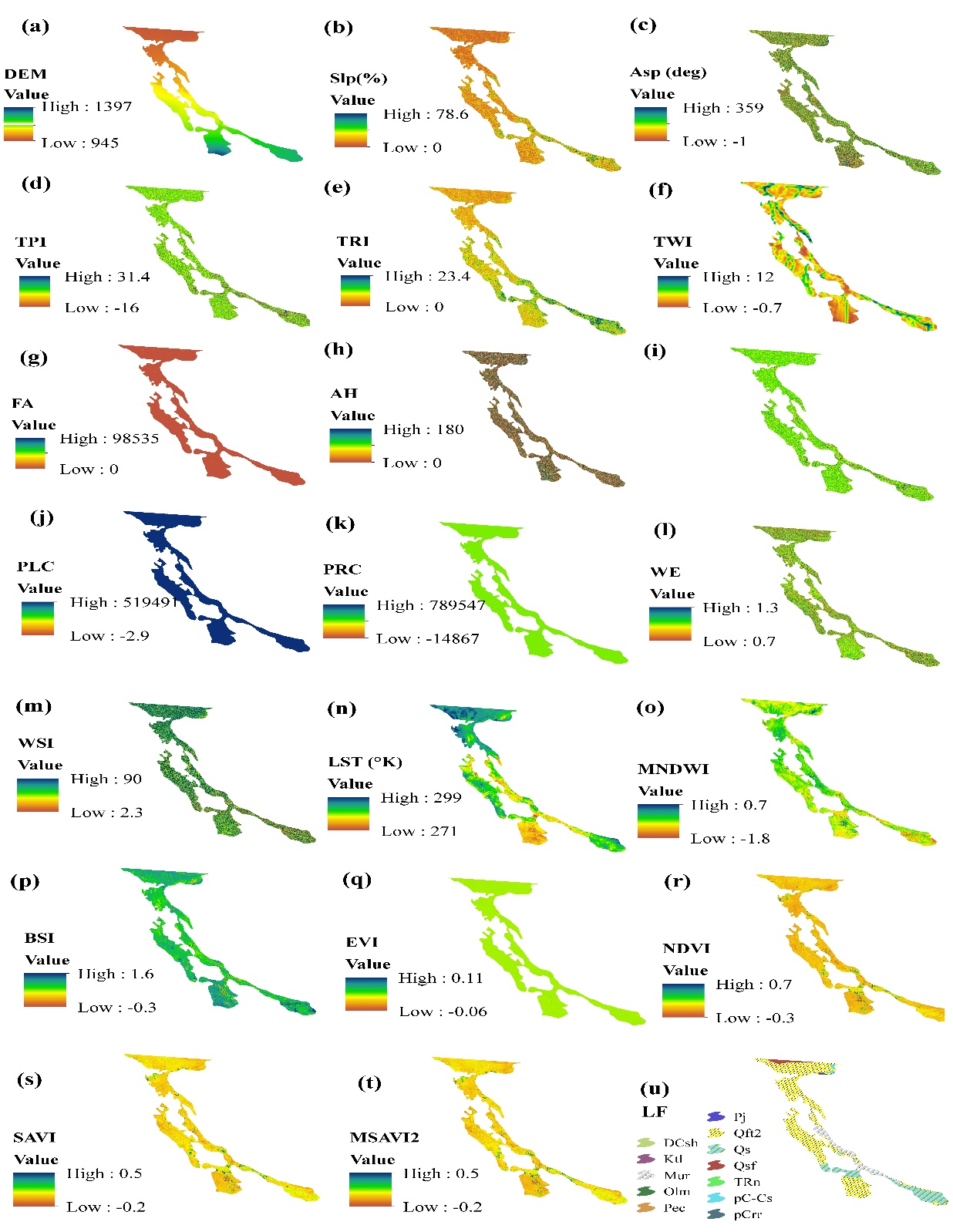


**Fig. S2.** Spatial distribution of land surface factors in the study area: (a) digital elevation model (DEM), (b) slope (Slp), (c) Aspect (Asp), (d) topographic position index (TPI), (e) terrain Ruggedness Index (TRI), (f) topographic wetness index (TWI), (g) flow accumulation (FA), (h) analytical hillshading (AH), (i)  longitudinal (Long), (j) plan curvature (PLC), (k) profile curvature (PRC), (l) wind exposition (WE), (m) wind shelter index (WSI), (n) land surface temperature (LST), (o) modified normalized difference water index (MNDWI), (p) bare soil index (BSI), (q) enhanced vegetation index (EVI), (r) normalized difference vegetation index, (s) soil adjusted vegetation index (SAVI), (t) modified soil adjusted vegetation index-2 (MSAVI2), (u) Lithological formations (LF): DCsh: Alternation of shale, marl and limestone, Ktl: Thin to medium bedded argillaceous limestone and thick bedded to massive, grey orbit Olina bearing limestone, Mur: Red marl, pestiferous marl, sandstone and conglomerate, Olm: Red and green silty, marl, sandstone and gypsum, Pec: Conglomerate and sandstone, Pj: Massive - bedded, dark - grey, partly reef type limestone and a thick yellow dolomite band in the upper part, Qft2: Low level pediment fan and terrace deposits, Qs: Sand dunes and sand sheet, Qsf: Salt flat, TRn: Sandstone, quartz arenite, shale and fossiliferous limestone, pC-Cs : Thick dolomite and limestone unit, portly cherty with thick shale intercalations, pCrr: Acidic volcanic rocks.


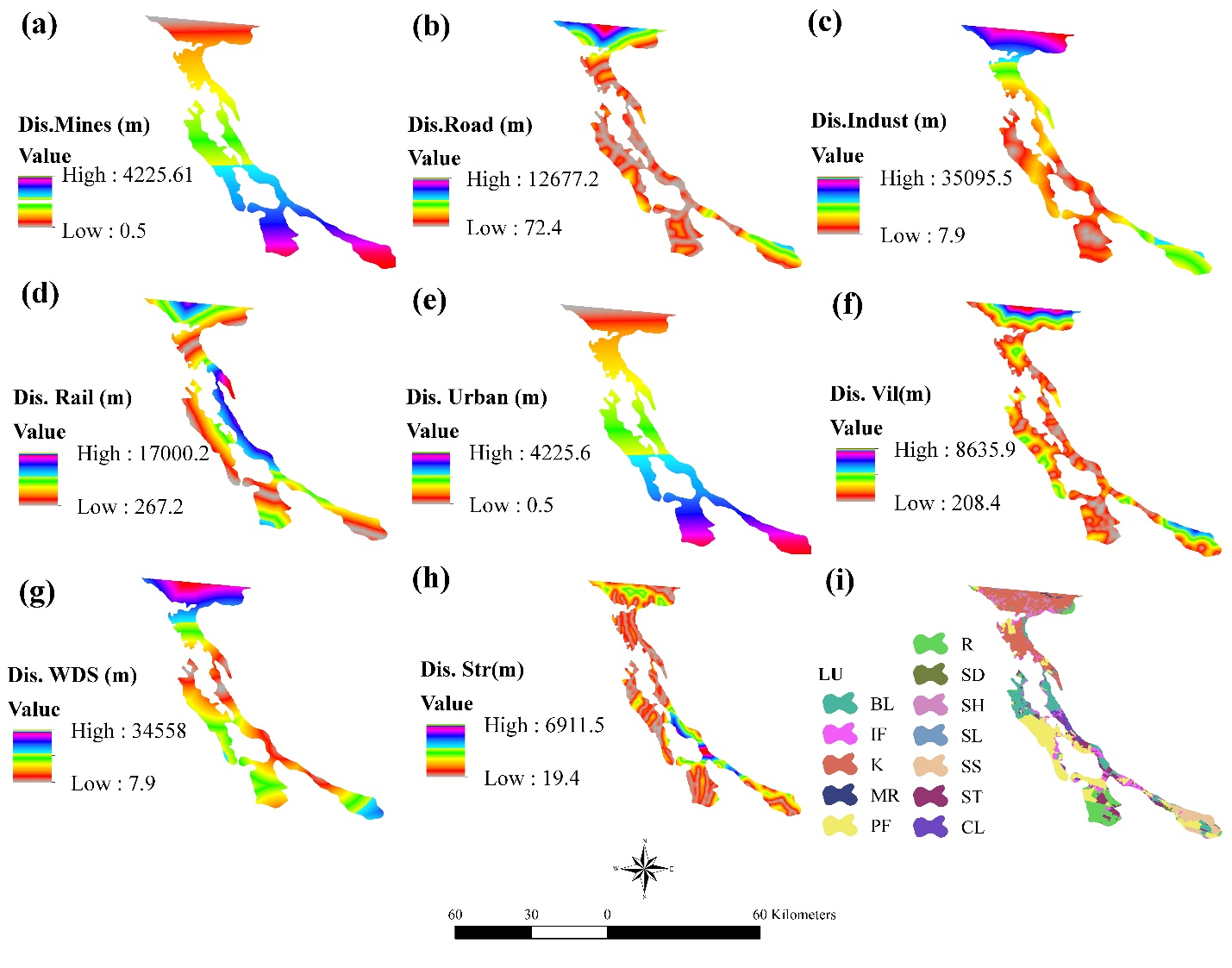


**Fig. S3.** Spatial distribution of human activity-based factors in the study area: (a) distance to mines (Dis.Mines), (b) distance to road (Dis.Road), (c) distance to industrial centers (Dis.Indust), (d) distance to railways (Dis.Rail), (e) distance to urban centers (Dis.Urban), (f) distance to villages (Dis.Vil), (g) distance to waste disposal sites (Dis.WDS), (h) distance to streams (Dis.Str), (i)  land use (LU). BL: Barren land, IF: Irrigated farming, K: Kavir, MR: moist regions, PF: planted forest, R: rangelands, SD: sand dunes, SH: Shrublands, SL: saline lands, ST: Settled terrain, and CL: clay lands.


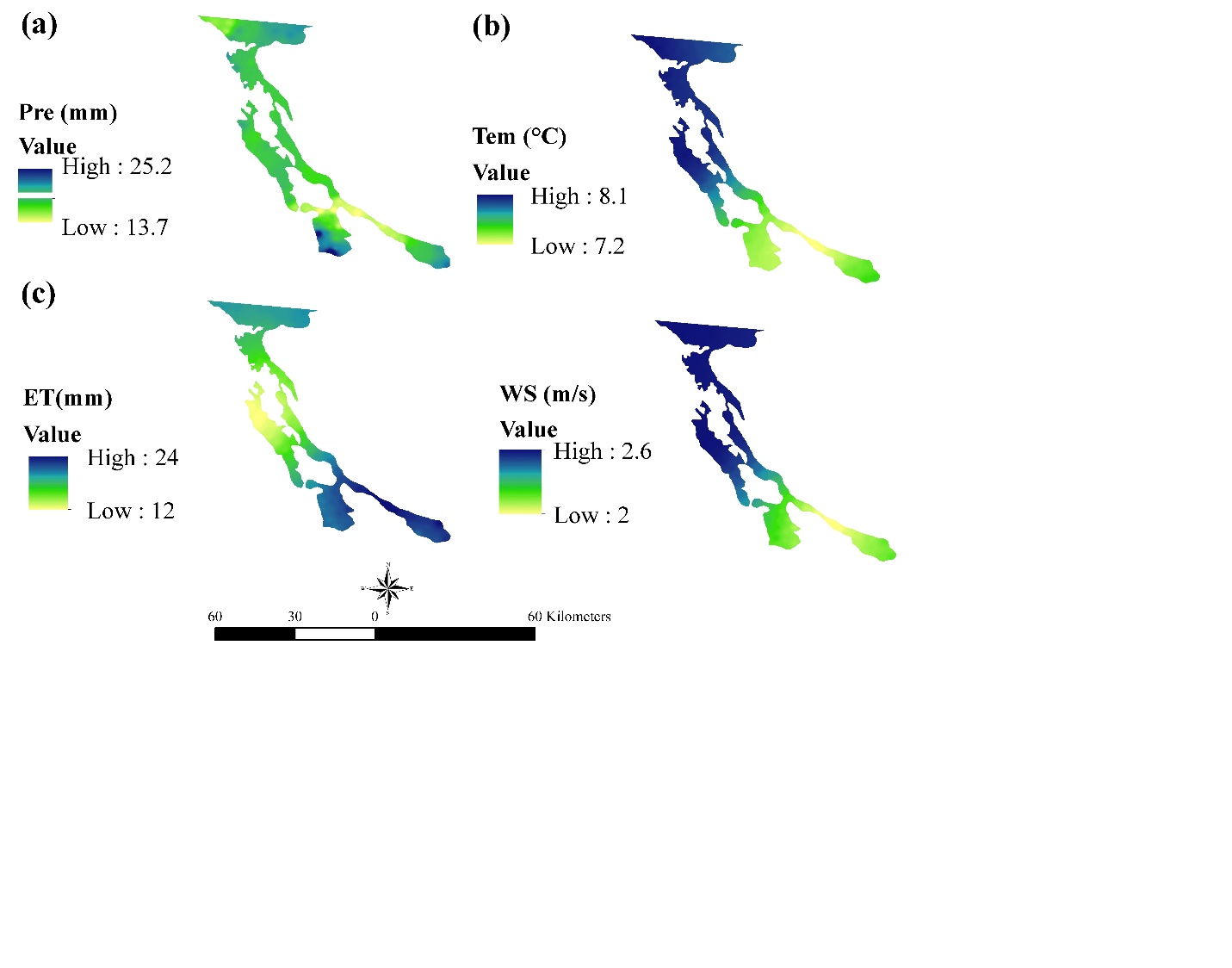


**Fig. S4.** Spatial distribution of meteorological factors in the study area: (a) precipitation (Pre), (b) temperature (Tem), (c) evapotranspiration (ET), (d) wind speed (WS).


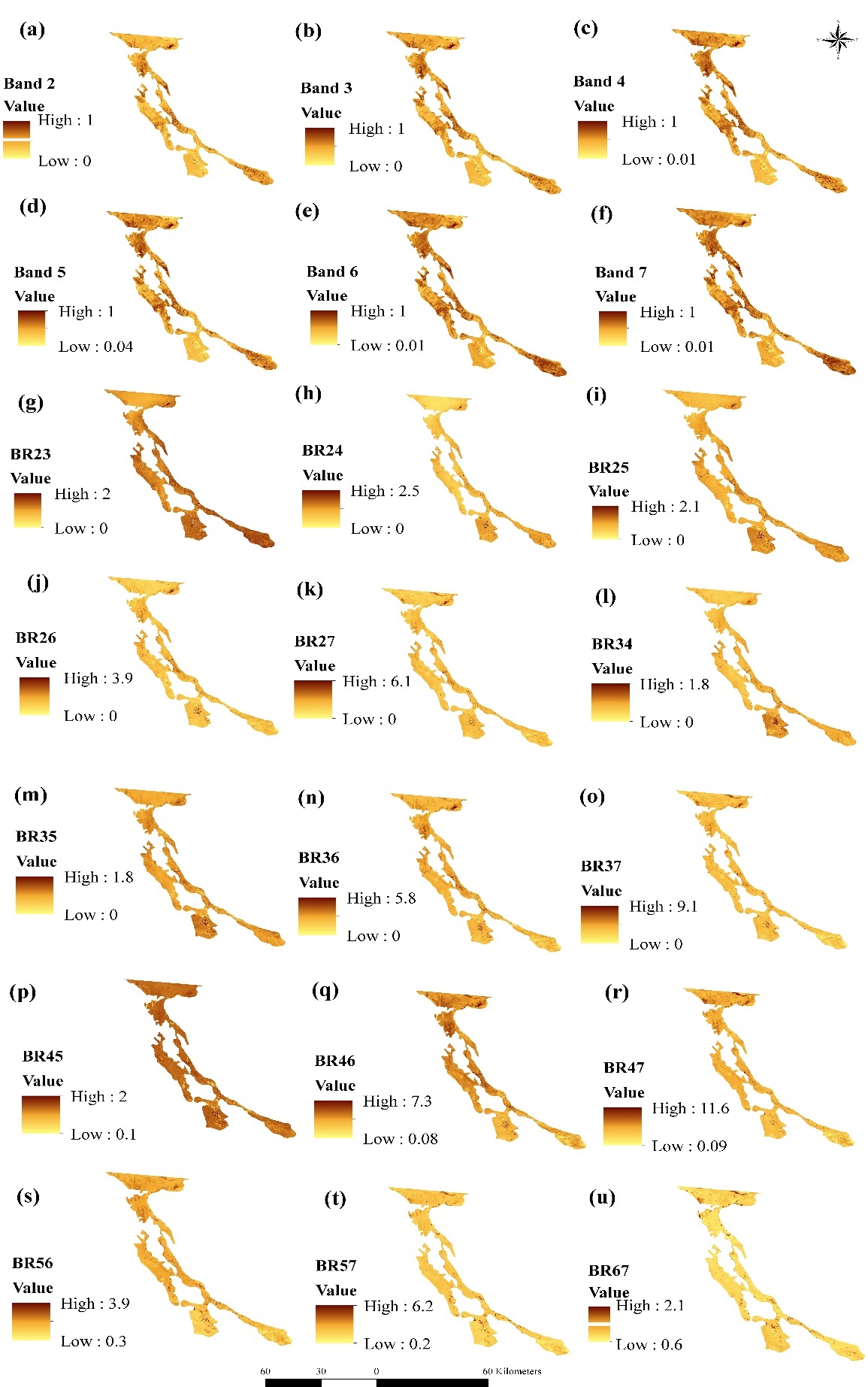


**Fig. S5.** The spectral bands (a–f) and their corresponding band ratios (g–u) for the study area. The maps illustrate the spatial distribution of values for Band 2 (a), Band 3 (b), Band 4 (c), Band 5 (d), Band 6 (e), Band 7 (f), and the ratios: Band Ratio 2/3 (BR23, g), Band Ratio 2/4 (BR24, h), Band Ratio 2/5 (BR25, i), Band Ratio 2/6 (BR26, j), Band Ratio 2/7 (BR27, k), Band Ratio 3/4 (BR34, l), Band Ratio 3/5 (BR35, m), Band Ratio 3/6 (BR36, n), Band Ratio 3/7 (BR37, o), Band Ratio 4/5 (BR45, p), Band Ratio 4/6 (BR46, q), Band Ratio 4/7 (BR47, r), Band Ratio 5/6 (BR56, s), Band Ratio 5/7 (BR57, t), and Band Ratio 6/7 (BR67, u).


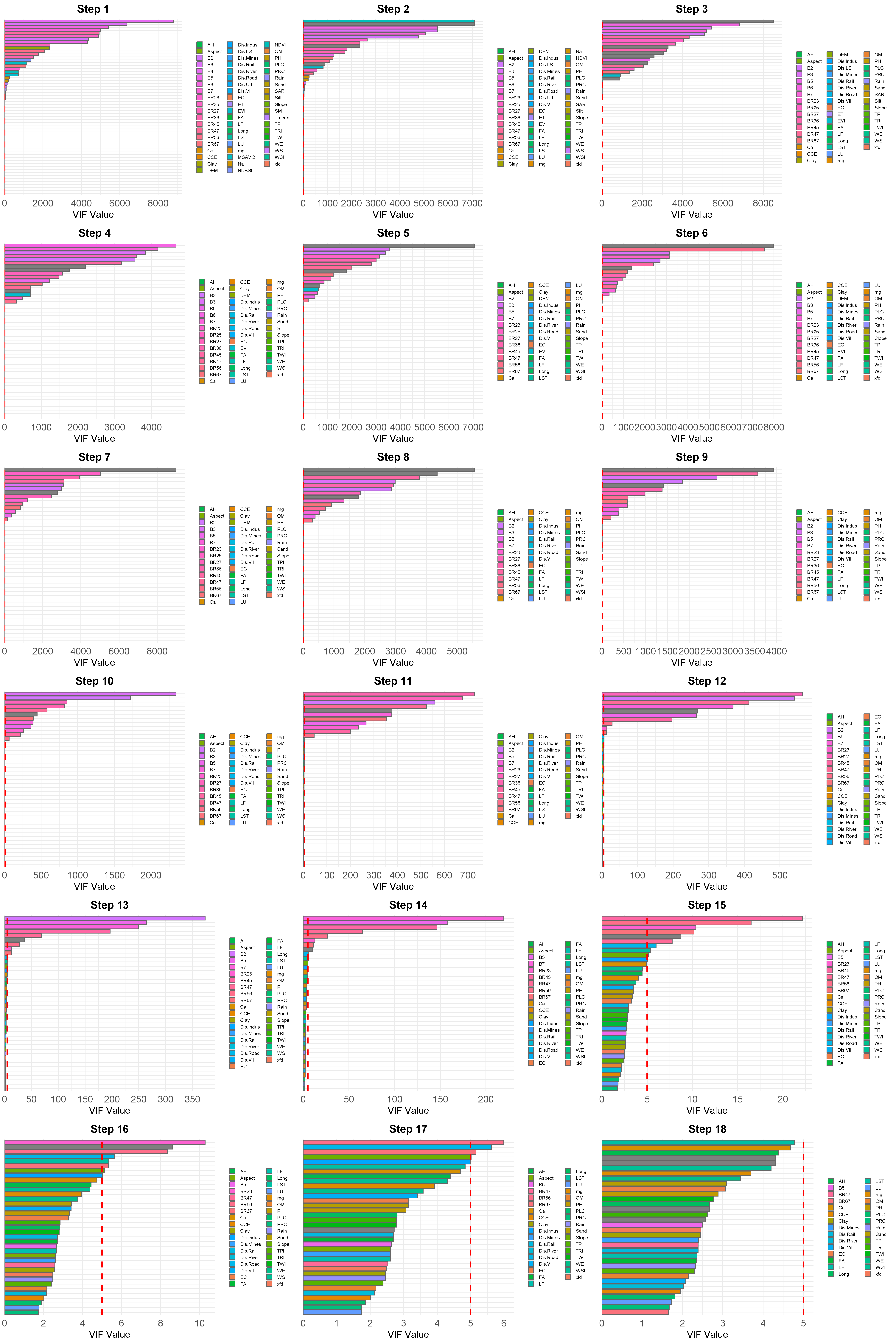


**Fig.S6.** Variance inflation factor values in different steps for selecting the optimal combination of environmental variables affecting soil PTEs in the study area.


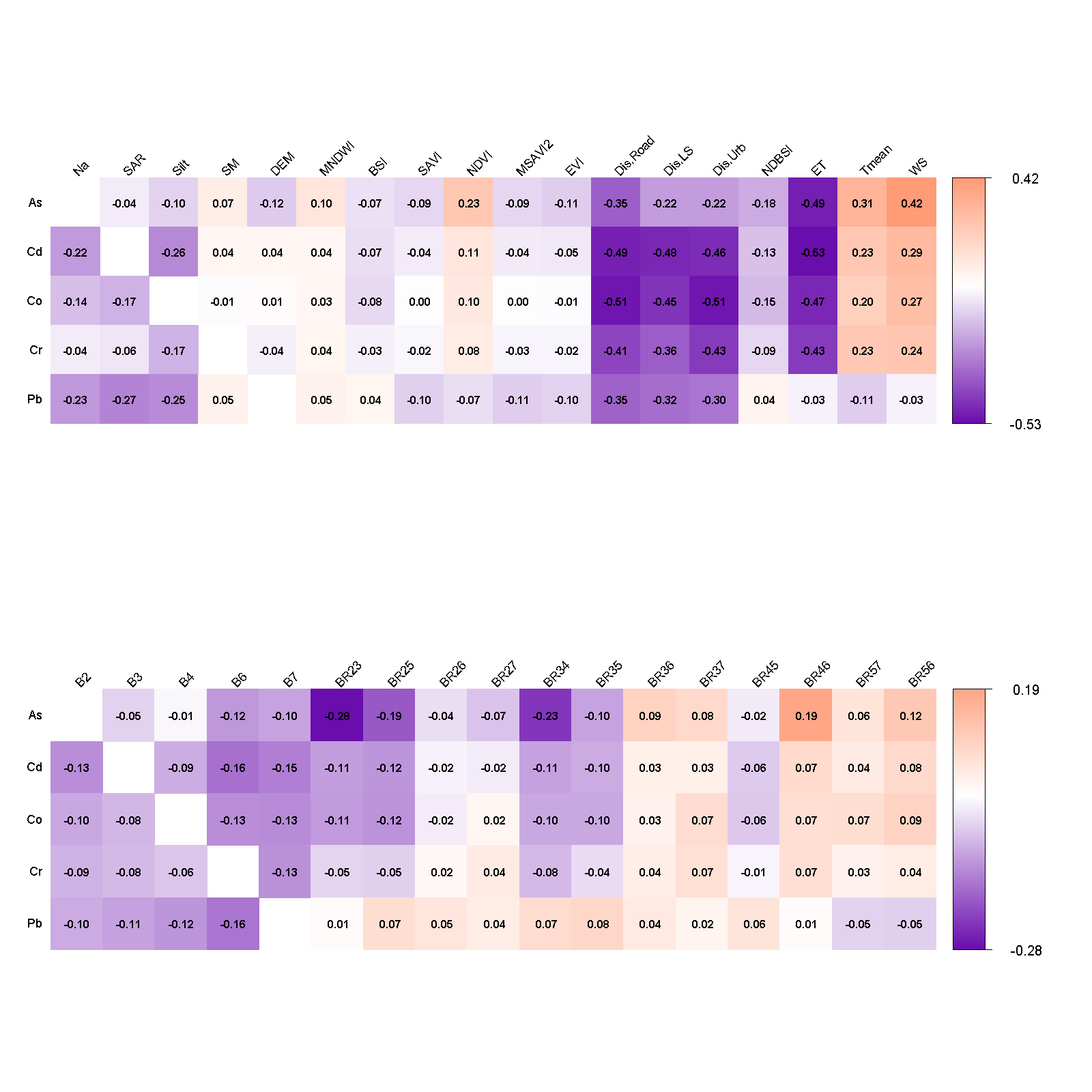


**Fig.S7.** Spearman correlation coefficients between environmental variables with the highest multicollinearity.

.


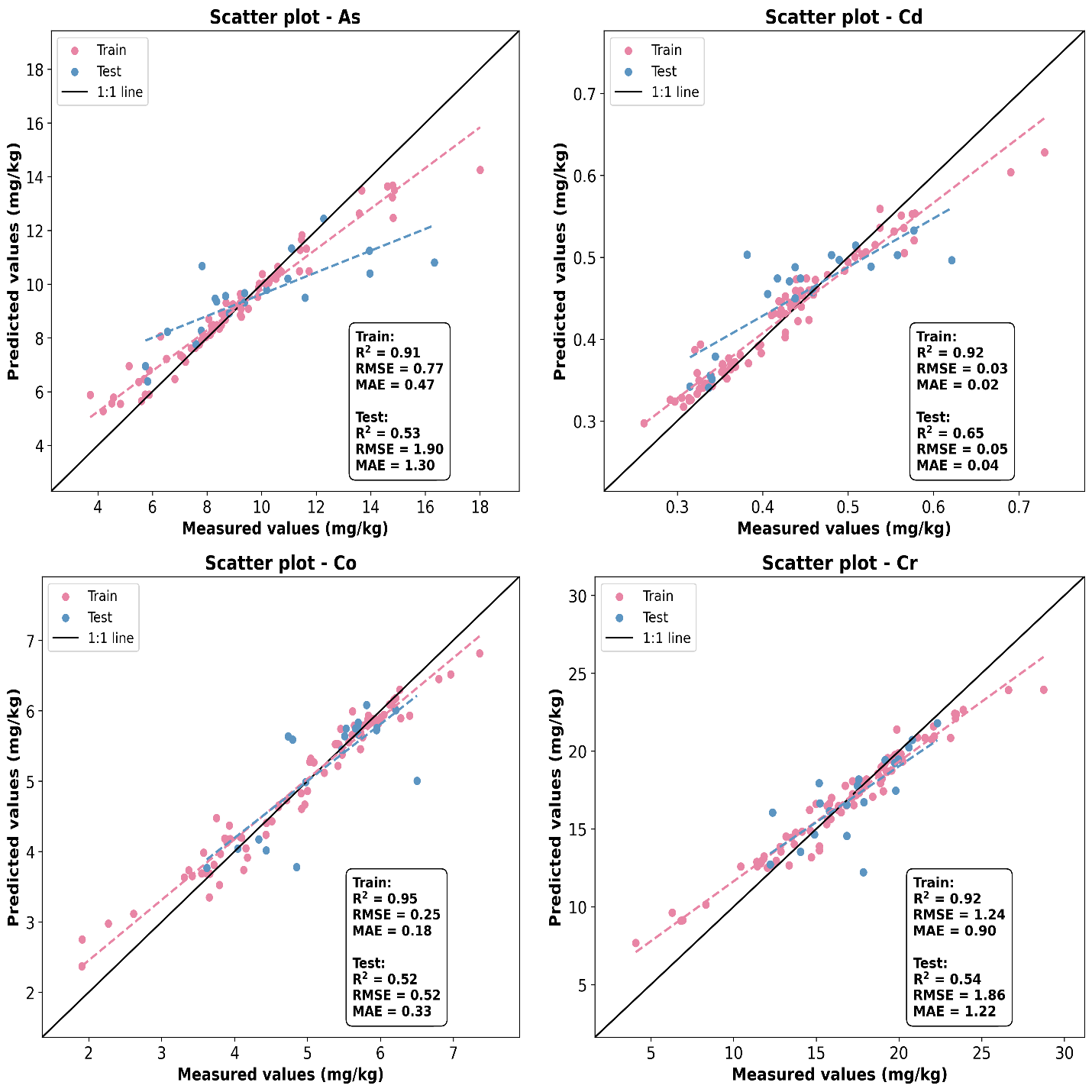


**Fig. S8.** Scatter plots of the measured concentrations against the predicted concentrations in training dataset and test dataset derived from RF model under optimal scenario for As, Cd, Co, and Cr.


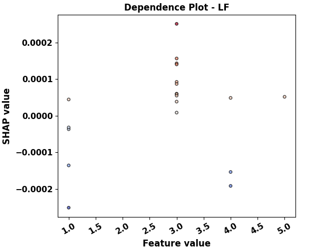


**Fig.S9.** SHAP Dependence Plot for the Lithological Formation (LF) variable. The numerical codes of 1, 2, 3, 4, and 5 represent Mur (Red marl, pestiferous marl, sandstone and conglomerate), pC-Cs (Thick dolomite and limestone unit, portly cherty with thick shale intercalations), Qft2 (Low level pediment fan and terrace deposits), Qs (Sand dunes and sand sheet), and Qsf (Salt flat), respectively.
